# A comparison of resting metabolic rate measurement in adults, using a ventilated canopy or a mixing‐chamber system with a silicone mask

**DOI:** 10.14814/phy2.70837

**Published:** 2026-03-25

**Authors:** Yair Pincu, F. N. U. Sakshi, Grant A. Chesbro, Akram Falahati, Rebecca D. Larson

**Affiliations:** ^1^ Department of Health and Exercise Science University of Oklahoma Norman Oklahoma USA; ^2^ Harold Hamm Diabetes Center University of Oklahoma Health Campus Oklahoma City Oklahoma USA; ^3^ TSET Health Promotion Research Center The University of Oklahoma Health Campus Oklahoma City Oklahoma USA; ^4^ Program for Cellular and Behavioral Neurobiology University of Oklahoma Norman Oklahoma USA

**Keywords:** indirect calorimetry, metabolic cart, total daily energy expenditure, ventilated hood

## Abstract

The most common method for measuring resting metabolic rate (RMR) via indirect calorimetry (IC) is using a ventilated canopy system (canopy). Yet, many use a facemask (mask). The purpose of this study was to compare RMR using a canopy and a mask. RMR was measured in 26 participants using a canopy or a mask, followed immediately by the opposite mode in a randomized fashion. RMR was compared between the canopy and mask in males and females using a 2 × 2 repeated‐measures ANOVA. Agreement between the 2 modes and typical error of measurement (TEM) were calculated. Data are mean ± SD. Resting VO_2_ and RER were significantly different (VO_2_ = 3.72 ± 0.70 vs. 3.46 ± 0.56 mL/kg/min; RER = 0.75 ± 0.04 vs. 0.80 ± 0.04 in canopy vs. mask, *p* < 0.05). RMR was lower using the mask (1719.71 ± 377.43 vs. 1791.23 ± 387.61 kcal/day, *p* = 0.026). Mask and canopy RMR showed poor agreement (95% Limit of agreement = −363.5 to 220.5 kcal/day). TEM was larger than the bias between the 2 methods (105.33 kcal/day), which suggests significant random error. Males (*n* = 14) had a larger and females (*n* = 12) smaller difference in RMR between canopy and mask (−79.14 ± 164.27, *p* = 0.063, vs. −62.62 ± 135.160 kcal/day, *p* = 0.166, respectively). We conclude that non‐ventilated facemask cannot produce comparable metabolic data and should not be used for RMR in place of a ventilated canopy.

## 1 INTRODUCTION

Energy balance is an important determinant of many metabolic diseases, including obesity and type 2 diabetes. It represents an equilibrium between the energy consumed and the energy expended by an individual. A positive energy balance, when one consumes more calories than expended, will result in weight gain, and if persisting over years and decades, will result in obesity and associated metabolic dysfunction. A negative energy balance, when energy expended is higher than energy consumed, will lead to weight loss and will promote improved metabolic function (Hill et al., 2012). Physical activity, the most modifiable component of daily energy expenditure, contributes only between 15% and 20% to daily energy expenditure (in sedentary and moderately active individuals, respectively). Resting Metabolic Rate (RMR), however, accounts for up to 70% of daily energy expenditure (Lam & Ravussin, 2016).

Accurate estimation of RMR using validated methods is important when evaluating total daily energy expenditure and when designing nutritional interventions, both in research and in the clinic. Routine evaluation of RMR is associated with greater weight loss in people with obesity undergoing weight loss intervention (Massarini et al., 2018). Underestimation of RMR could lead to inadequate caloric consumption and malnutrition which can be detrimental for critically ill patients (Ndahimana & Kim, 2018), or lead to undesired weight loss, inadequate recovery, and decreased performance in athletes, a phenomenon termed relative energy deficiency in sports (REDs) (Ackerman et al., 2023). Alternatively, overestimation of RMR could lead to overconsumption, weight gain, and increased cardiometabolic disease risk (Hill et al., 2012).

The most common method for measuring RMR is indirect calorimetry, in which expired O_2_ and CO_2_ are measured, and caloric expenditure is calculated, typically using the modified Weir equation (Weir, 1990). The most common indirect calorimetry method to measure RMR is using a ventilated canopy system (canopy) with a dilution pump (Schoffelen & Plasqui, 2018). This system uses an external pump that maintains a constant rate of air flow through the canopy (typically 20–40 L/min), which dilutes the expired air with room air within the canopy, before it is collected into a mixing chamber and analyzed for gas composition. However, in some cases, when access to a ventilated canopy system is limited, the use of a non‐ventilated silicone mask (mask) for evaluating RMR has become common (Črešnovar et al., 2025; Güzel et al., 2025; Liang et al., 2025; Niclou et al., 2025).

Although several studies have compared RMR measured using non‐ventilated methods with RMR measured using canopy systems, these yielded inconsistent findings (Isbell et al., 1991). Some of these studies were done many years ago (McAnena et al., 1986; Segal, 1987) used older mask designs (Forse, 1993; Segal, 1987) or mouthpieces (Forse, 1993; Roffey et al., 2006), or compared between different indirect calorimetry instruments (Compher et al., 2006; Dupertuis et al., 2022; Wang et al., 2017). While some studies reported high reliability and validity of the mask compared to the canopy system (Isbell et al., 1991; McAnena et al., 1986), others (Graf et al., 2013; Roffey et al., 2006) reported varying magnitudes of differences in RMR between the two methods. A modern silicone mask has been routinely used in indirect calorimetry systems for the past 15 years or so. It is possible that improvements in mask design and materials in the modern silicone mask might reduce these differences. Such studies, directly comparing RMR measurement using a canopy and a modern silicone mask in the same instrument, are scarce. Additionally, very few studies compared the differences in RMR measurement using the 2 methods between males and females. It is not clear if modern silicone masks that are ubiquitously used today can be used for RMR measurement in place of ventilated canopy systems in the same instrument.

Therefore, the purpose of this study is to methodically compare RMR measurement via indirect calorimetry using a canopy and a mask in males and females in the same metabolic system. We hypothesize that RMR will not be significantly different between the 2 testing modes.

## 2 METHODS

The study was reviewed and approved by the University of Oklahoma Institutional Review Board and is in accordance with the Declaration of Helsinki. All participants gave their written informed consent prior to participating in the study.

### 2.1 Participants

Thirty‐six adult participants of both sexes were recruited for the study. Sample size calculation was performed based on power analysis and is detailed in the statistical analysis section. Inclusion criteria were age 18–65 years with no active chronic or acute disease. Exclusion criteria were smoking or taking cardiovascular or metabolism‐altering medications. Participants were instructed to arrive at the lab between 7 and 8 am, following an overnight fast, having abstained from any food, sugary drinks, alcohol, or caffeine for at least 8 h. Participants were also instructed to refrain from moderate or vigorous physical activity for 24 and 48 h before testing, respectively.

### 2.2 Experimental design

In this crossover repeated measures study, participants visited the lab once and were subjected to RMR measurement using a canopy or a mask, followed immediately by a second RMR measurement using the opposite mode. The order of canopy and mask was randomized. After the completion of the RMR measurement, height, weight, and waist circumference were measured. All measurements were conducted in a temperature‐controlled room with an ambient temperature of 21°C–23°C, and the subjects were covered with a light fleece blanket.

### 2.3 RMR measurement

RMR was measured in adherence to the recommendations of the Evidence Analysis Working Group of the American Dietetic Association (Compher et al., 2006). In brief, following a 15–20 min resting period and set up, participants were asked to lie supine with their head resting on a soft pillow. They were asked to be still for approximately 40 min while expired air was collected into a metabolic cart (TrueOne 2400, ParvoMedics, Inc., Salt Lake City, UT) for analysis using a silicone facemask (7450 V2 ORO‐Nasal Reusable Face Mask, Hans Rudolf, Shawnee, KS) and for an additional 40 min while using a ventilated canopy system (ParvoMedics, Inc., Salt Lake City, UT). The metabolic cart was calibrated every morning, according to the manufacturer's instructions.

When the ventilated canopy system was used, the hood was placed over the head of the participant, and the canopy was draped around the torso and tucked to minimize air leaks. The flow rate in the dilution pump was adjusted during the first 5 min of the test so that the fraction of expired CO_2_ (FeCO_2_%) was between 1.0% and 1.2% and was maintained throughout the test. The following 35‐minute period was recorded and used for data analysis.

The silicone mask was used in the RMR mode software, without the dilution pump and using a mixing chamber (not a breath‐by‐breath system). A two‐way non‐rebreathing T‐shape valve (Hans Rudolf, Shawnee, KS) was used to connect the mask to the metabolic cart tubing. After the mask was fitted on the participant's face, they were instructed to lie supine, and the measurement started. The first 5 min of the measurement were discarded, and the following 35 min period was recorded and used for data analysis.

RMR was determined in a two‐step process. First, a visual inspection of the graph plotting RMR over time identified at least a 5‐min segment in which RMR was most stable. Then, data for that segment (in 30‐s intervals) were averaged, and the coefficient of variance (CV) was calculated to determine the steady state (CV ≤10%). If a steady state was not achieved (CV >10%), the measurement was discarded. If a participant had a discarded RMR measurement in either the mask or canopy mode, both RMR measures were disqualified, and that participant was excluded from the study.

### 2.4 Statistical analysis

Sample size was determined using power calculation (G*Power 3.1) based on previous work that compared RMR between canopy and mask in the same instrument (Graf et al., 2013). For a mixed model repeated measures ANOVA to detect RMR difference of approximately 100 kcal with a moderate effect size (*f* = 0.165), power of 80%, and *α* = 0.05, a minimum sample size of *n* = 26 was needed. Therefore, a sample size of 36 was recruited. The difference in RMR (∆RMR) between the 2 testing modes was calculated by subtracting the canopy from the mask. The effects of testing mode and sex on VO_2_, VCO_2_, RER, and RMR were tested using a mixed model 2 × 2 repeated measures ANOVA with pairwise comparisons using the Bonferroni adjustment for multiple comparisons. Testing mode (mask vs. canopy) was the within‐subject factor, and Sex (male vs. female) was the between‐subject factor. Effect sizes were determined using partial eta squared (ηp^2^), where an effect of 0.01–0.05 indicated a small effect, 0.06–0.13 indicated a moderate effect, and over 0.14 indicated a large effect size (Cohen, 1988). For effect sizes of paired *t*‐tests, ηp^2^ was calculated as *t* ^2^/*t* ^2^ + df. ICCs were used to assess the parallel‐test reliability of the mask vs. canopy system, and Bland–Altman analyses (Giavarina, 2015) were conducted to determine the bias and limits of agreement between canopy and mask RMR, in the entire sample and the different sexes. ICCs and their 95% confidence intervals were calculated based on a two‐way mixed‐effects, single measure (*k* = 1), absolute agreement model. An ICC of <0.50 indicated poor parallel‐test reliability, 0.51–0.75 indicated moderate parallel‐test reliability, 0.76–0.90 indicated good parallel‐test reliability, and >0.90 indicated excellent parallel‐test reliability (Koo & Li, 2016). As a secondary analysis of reliability typical error of measurement (TEM) for RMR was calculated as the standard deviation of the RMR difference between canopy and mask divided by square root of 2 measurements and was expressed in absolute units and as a percentage of the average mean RMR of both methods. TEM provides an estimate of the random measurement error (precision) associated with repeated measurements within individuals and is recommended as a measure of absolute reliability in repeated‐measure designs (Atkinson & Nevill, 1998; Hopkins, 2000). Mean group effects on RMR between mask and canopy in males with and without facial hair were tested using a group by time repeated measure ANOVA and Student's *t*‐test was used to compare age, body weight and BMI between males with and without facial hair. Mann–Whitney *U* test was used to test the effect of testing order (mask first vs. canopy first) on ∆RMR. Statistical significance was set at *p* ≤ 0.05.

## 3 RESULTS

### 3.1 Participants

Out of 36 participants recruited, five were excluded due to a lack of at least five consecutive minutes of RMR steady state (2 only in the mask, 3 in both the mask and canopy). An additional five participants were excluded for the following reasons: erroneous RMR (*n* = 2, mask RMR <1000 kcal/day, smaller than 50% of expected RMR by body weight), being an extreme outlier (over 3 standard deviations of the mean, *n* = 2), or due to data loss from a corrupted file (*n* = 1). The analysis was conducted in a final sample size of *n* = 26. Data are presented as mean ± SD, unless otherwise stated.

All participants were healthy, with no obesity or any cardiometabolic disease (Table 1). Mean age was 25.3 ± 6.7 years, and BMI was 26.3 ± 5.7 kg/m^2^. A total of 14 males and 12 females were analyzed. Of the male participants, 50% had facial hair.

**TABLE 1:** Participants’ characteristics.

	All, *n* = 26	Males, *n* = 14	Females, *n* = 12	*p*‐value
Age (years)	25.3 ± 6.7	26.4 ± 5.6	24.0 ± 7.8	*p* = 0.349
BMI (kg/m^2^)	26.3 ± 5.7	27.4 ± 6.4	24.9 ± 4.5	*p* = 0.280
Body weight (kg)	73.2 ± 17.7	82.6 ± 17.2	62.2 ± 10.9	***p* < 0.001**
Waist circumference (cm)	86.5 ± 14.1	93.6 ± 13.4	78.2 ± 10.2	***p* = 0.003**
Systolic BP (mmHg)	112.9 ± 11.7	118.4 ± 8.4	106.4 ± 11.9	***p* = 0.006**
Diastolic BP (mmHg)	77.5 ± 6.7	78.8 ± 7.2	75.9 ± 6.1	*p* = 0.287
MAP (mmHg)	89.3 ± 7.5	92.0 ± 7.2	86.0 ± 6.8	***p* = 0.043**
Bearded^a^ [*n* (%)]	7 (26.9%)	7 (50.0%)	0 (0%)	***p* = 0.004**
Mask First^a^ [*n* (%)]	9 (34.6%)	4 (28.6%)	5 (41.7%)	*p* = 0.484

*Note*: Data are mean ± SD. Sex differences in Age, BMI, body weight, waist circumference, and blood pressure were tested using Student's *t*‐test. Bold indicates significant *p* values.

^a^ Sex differences in Bearded and Mask First were tested using a chi‐squared test.

### 3.2 Metabolic measures

Rates of oxygen consumption (VO_2_) were significantly lower when measured using the mask compared to the canopy (∆ = −0.26 mL/kg/min, *F* = 9.506, *p* < 0.01, ηp^2^ = 0.28; Table 2), while rates of CO_2_ production (VCO_2_) were not different (∆ = −0.01 mL/kg/min, *F* = 0.007, *p* = 0.933, ηp^2^ = 0.000). RER, the ratio between VCO_2_ and VO_2_, which is indicative of substrate utilization, was significantly higher when measured using the mask compared to the canopy (∆ = −0.05, *F* = 39.072, *p* < 0.001, ηp^2^ = 0.62). Differences in RER and lack of differences in VCO_2_ measures between the testing modes were consistent across the sexes, but differences in VO_2_ were not statistically significant in females (Table 2).

**TABLE 2:** Metabolic measures.

Test mode	Ventilated canopy			Silicone mask		
	VO_2_	VCO_2_	RER	VO_2_	VCO_2_	RER
	mL/kg/min	mL/kg/min		mL/kg/min	mL/kg/min	
All (*n* = 26)	3.72 ± 0.70	2.78 ± 0.51	0.75 ± 0.04	3.46 ± 0.56^a^	2.77 ± 0.51	0.80 ± 0.04^b^
Males (*n* = 14)	3.71 ± 0.78	2.81 ± 0.66	0.75 ± 0.05	3.40 ± 0.51^a^	2.72 ± 0.51	0.80 ± 0.05^b^
Females (*n* = 12)	3.73 ± 0.63	2.74 ± 0.41	0.74 ± 0.03	3.52 ± 0.63	2.81 ± 0.54	0.80 ± 0.04^b^

*Note*: Data are mean ± SD. Main effects of Test Mode and Sex and Test Mode by Sex interaction were tested using repeated measures ANOVA with Test Mode as the within‐subject and Sex the between‐subject factors. Differences between Test Modes within each Sex were tested using pairwise comparisons with Bonferroni adjustments for multiple comparisons.

^a^ *p* ≤ 0.05.

^b^ *p* ≤ 0.001 compared to Ventilated Canopy.

### 3.3 Parallel‐test reliability and agreement between mask versus canopy

**FIGURE 1:**
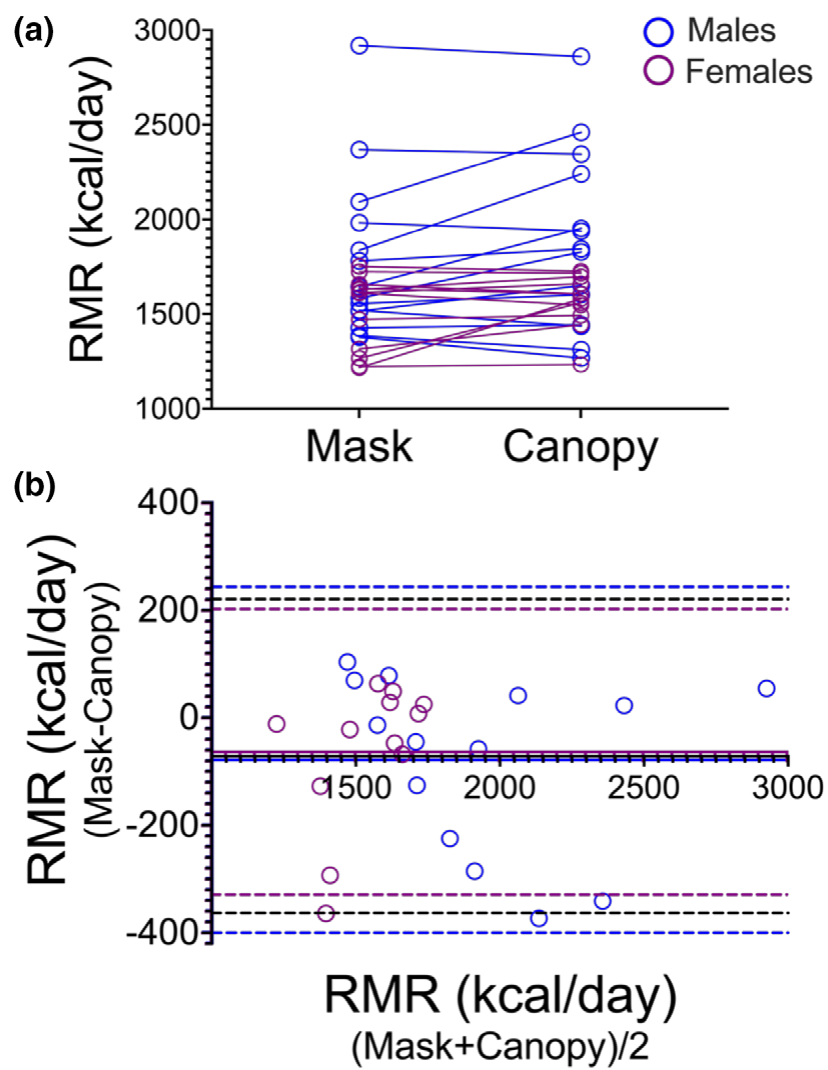
Comparison between RMR using Mask and Canopy. Individual RMR values (kcal/day) in mask and canopy (a) and A Bland–Altman plot of agreement between mask and canopy (b) are presented. Blue circles represent males and purple circles represent females. In the Bland–Altman plot (b), dashed horizontal lines represent mean bias and limits of agreement for the entire sample (Black, −71.52 kcal/day, −363.5 to 220.5 kcal/day), males (Blue, −79.14 kcal/day, −401.1 to 242.8 kcal/day), or females (Purple, −62.62 kcal/day, −328.4 to 203.1 kcal/day).

Mean RMR values were significantly lower when measured using the mask compared to the canopy (*F* = 5.63, *p* < 0.05, ηp^2^ = 0.19, Table 3), but there was large inter‐individual variability in ∆RMR between the mask and the canopy (Figure 1a). Standard deviation of ∆RMR was more than double the mean difference between the 2 methods (Table 3). This resulted in CV = 208% which suggests significant random measurement error.

**TABLE 3:** Differences in RMR between Ventilated Hood and Silicone Mask.

	RMR (kcal/day)			Test mode main effect	Effect size
	Ventilated canopy	Silicone mask	Delta		
All (*n* = 26)	1791.23 ± 387.61	1719.71 ± 377.43	−71.52 ± 148.97	***p* = 0.026**	0.190
Males (*n* = 14)	1978.73 ± 436.82	1899.59 ± 405.29	−79.14 ± 164.27	*p* = 0.063	0.137
Females (*n* = 12)	1572.47 ± 137.87	1509.85 ± 201.00	−62.62 ± 135.60	*p* = 0.166	0.078
Sex main effect	***p* = 0.005**	***p* = 0.006**	Test Mode by Sex interaction	*p* = 0.784	

*Note*: Data are mean ± SD. Main effects of Test Mode and Sex and Test Mode by Sex interaction were tested using repeated measures ANOVA with Test Mode as the within‐subject and Sex the between‐subject factors. Differences between Test Modes within each Sex were tested using pairwise comparisons with Bonferroni adjustments for multiple comparisons. Effect size is reported using Partial Eta Square (ηp^2^). Bold indicates significant *p* values.

Nevertheless, ICC for the entire sample demonstrated excellent reliability between the measures with ICC coefficient = 0.924 (95% CI = 0.838–0.965. *p* < 0.001). In the male participants, reliability was excellent (ICC = 0.924, 95% CI: 0.781–0.975, *p* < 0.001), while in the female participants, moderate reliability was observed (ICC = 0.691, 95% CI: 0.223–0.900, *p* = 0.004). Bland–Altman analyses of agreement (Figure 1b) showed a moderate systematic bias but no proportional bias, and wide limits of agreement (LOA) between the measurements (independently of sex). For the entire sample, the systematic bias in RMR between the mask and canopy was −71.52 ± 148.97 with 95% LOA between −363.5 and 220.5 kcal/day. Similar values were calculated for males (bias = −79.14 ± 164.27, 95% LOA = −401.1 and 242.8 kcal/day) and females (bias = −62.62 ± 135.60, 95% LOA = −328.4 and 203.1 kcal/day). Interestingly, the biases for the entire sample and for each of the sexes were all within the 95% confidence interval of day‐to‐day variation in RMR previously reported (79.2 kcal/day, 95% CI = 55.8–102.6 kcal/day) (Haugen et al., 2003). However, the wide LOA (±292.02, ±321.94, and ± 265.72 kcal/day for the entire sample, for males, and for females, respectively) considerably exceeded the reported day‐to‐day variability of RMR, indicating poor agreement for individual‐level assessment.

TEM relative to the mean RMR of the 2 methods for the entire sample was moderate and was not affected by sex (TEM = 6.0%, 5.98%, and 6.22% for the entire sample, males and females, respectively). Absolute TEM for RMR between the 2 methods was 105.3 kcal/day for the entire sample, 116.2 kcal/day for males, and 95.9 kcal/day for females. These values, as well, exceed previously reported RMR day‐to‐day variability, suggesting that the random error of measurement and disagreement between the mask and canopy exceed the magnitude of typical biological day‐to‐day fluctuations in RMR and that using the mask instead of the canopy could introduce variability greater than real physiological change in RMR.

### 3.4 Effects of facial hair in males

Facial hair can affect the fit and seal of the face mask. Despite the fact that the mask fit and air seal were tested before each measurement, we tested the effect of facial hair on the differences in RMR measurements between the two methods in males. No significant differences were detected in RMR between males with or without facial hair, independently of testing mode (*F* = 4.40, *p* = 0.058, ηp^2^ = 0.268; RMR = 1924.34 ± 551.40 vs. 1874.84 ± 224.56 kcal/day, in males with vs. without facial hair in the mask, and 1914.34 ± 540.23 vs. 2043.12 ± 334.50 kcal/day, in males with vs. without facial hair in the canopy, respectively). However, a significant interaction was detected between the testing mode and facial hair (*F* = 5.573, *p* = 0.036, ηp^2^ = 0.317). Pairwise comparisons reveal that while RMR was not different between the mask and canopy in males with facial hair (mean difference in RMR = 10.00 ± 79.28 kcal/day, *p* = 0.854, ηp^2^ = 0.003), in males without facial hair, RMR was significantly lower when measured using the mask compared to canopy (mean difference in RMR = −168.28 ± 183.39 kcal/day, *p* = 0.08, ηp^2^ = 0.453). Absolute TEM for RMR between the 2 methods was 56.07 kcal/day for males with facial hair and 129.67 kcal/day for males without facial hair. TEM relative to the average mean RMR between the 2 methods for males without facial hair was moderate (6.62%) and similar to that of the entire sample. However, for males with facial hair relative TEM was 2.92%, which is considered very good. No significant differences between males with and without facial hair were found in age (*t* = 2.02, *p* = 0.07, ηp^2^ = 0.25), body weight (*t* = 1.26, *p* = 0.23, ηp^2^ = 0.18), or BMI (*t* = 0.64, *p* = 0.53, ηp^2^ = 0.03). It seems that in this study, facial hair was not associated with increased random measurement error in RMR and that differences in RMR between males with and without facial hair represent a type 1 error, likely due to the small sample size (*n* = 7 per group).

### 3.5 Effect of testing order

While the testing order was randomized, due to the exclusion of 10 participants (8 of which were tested using the mask first), a greater proportion of participants in the remaining sample were tested using the ventilated canopy first (Table 1). This prompted us to test if the order of testing affected the difference between RMR using the mask vs. the canopy. We found no significant differences in ∆RMR between the two groups (−56.43 ± 147.90 kcal/day, mean rank = 14.33 vs. −79.50 ± 153.43 kcal/day, mean rank = 13.06 in mask first (*n* = 9) vs. canopy first (*n* = 17), *U* = 69.0, *p* = 0.711).

## 4 DISCUSSION

In this study, we used a cross‐over repeated measure approach in a combined male and female sample to compare RMR measurement using a ventilated canopy and a commonly used Hans Rudolf silicone mask in a randomized order. RMR was measured sequentially (in the same visit) using the two testing modes in the same metabolic cart.

Since indirect calorimetry using a ventilated canopy is regarded by many as the gold standard for RMR measurement, we tested the reliability of the non‐ventilated silicone mask compared to the canopy method. We found that RMR was significantly underestimated (by 71 kcal/day) using the mask compared to the canopy, and that the agreement between the two methods was poor with extreme limits of agreement (±292.02 kcal/day). There was also an excessively large variability in ∆RMR (CV = 208%) and a large random error. We did not find an effect of sex on the measures of reliability between the 2 methods.

Our results suggest that while the mask and canopy are both consistent in ranking individuals according to their RMR and can distinguish between participants with high and low RMR, the poor agreement between the methods suggests that the 2 methods cannot be used interchangeably. The limits of agreement between −363.5 and 220.5 kcal/day would suggest that for a given individual the mask could overestimate or underestimate RMR by approximately 300 kcal/day which is unacceptable.

Our results also show that there was a typical error of measurement of about 105 kcal/day or 6% of the mean overall RMR. While a 6% error is relatively low and suggests that the RMR measure is stable, the massive CV of the difference and the very wide limits of agreement point to a very low signal‐to‐noise ratio likely due to large random error of measurement. Evidently, the mean difference between the methods is much smaller than the measurement error. Although we cannot determine the source of the error of measurement, it might be due to differences in air collection between the 2 methods or perhaps due to differences in dead space in the canopy system and the mask and two‐way non‐rebreathing valve system. However, since a large component of the error in this study was likely random, this explanation might not be sufficient. We also considered that the large variability in RMR and in ∆RMR might be due to the large range of body weights in our sample. Body weights ranged between 47 and 122 kg, which resulted in a wide range of RMR as well, between 1227.7 and 2926.3 kcal/day, and variability was also similar between the 2 variables (CV = 24.2 and 21.4% for body weight and overall RMR, respectively). However, there was no correlation between body weight and ∆RMR (*r* = 0.07, *p* = 0.71), suggesting no effect of body weight on the difference in RMR between the 2 methods.

Since 5 of the original 36 participants recruited for the study were excluded due to failure to achieve a steady state (CV>10%), and of these 5, all did not reach steady state in the mask, we suspect that in some instances there might have been some air leakage from the mask that introduced additional error. We did not find significant differences in any of the participants' characteristics (age, BMI, Body weight, Waist circumference, or Blood pressure measures) between the participants that were included and the ones that were excluded from the study (data not shown).

Our findings are in agreement with several papers that reported significant differences between mask and canopy and recommend practicing caution when planning to use a mask in place of a ventilated canopy for RMR measurement. However, when considering findings from these papers together, it gives stronger support to our results. While differences between mask and canopy were reported, compared to the canopy, the mask either overestimated RMR by 118 kcal (Forse, 1993), or underestimated RMR by 115 kcal (Graf et al., 2013). Interestingly, Bland–Altman analysis of agreement between RMR measured using a mask or a canopy yielded similar bias to what we report here, but unlike our work, Dupertuis et al. (2022) reported 73 kcal/day overestimation of RMR by the mask compared to the canopy. Similar to our findings, LOA were also very wide (−411 to 557 kcal/day) (Dupertuis et al., 2022).

While other studies report no difference in RMR between mask and canopy, RMR was only reported as a percent of predicted daily energy expenditure for the paired analysis (McAnena et al., 1986), or a group design was used whereby each testing mode was conducted on a separate group (women only), and no participant characteristics were reported (Isbell et al., 1991), which limits the interpretation of these findings in comparison with our work. Moreover, unlike our design, in which a single metabolic system was used for both testing modes, McAnena et al. (1986), and Dupertuis et al. (2022), used a different instrument for each testing mode, thus compounding the error with the variability between the two systems.

### 4.1 Study limitations

We recognize several limitations to our work. First, the study was not conducted in a clinical center, and the participants did not spend the night in the lab nor was the RMR measurement conducted as soon as the participants woke up. This might introduce some error to the measurement due to a certain amount of energy expended during morning routine and ambulation to the lab. In order to account for that, all participants were rested for about 20 min before beginning the RMR test and were instructed to arrive to the lab fasted with as little walking as possible. Second, we did not assess test–retest reliability of each testing mode by assessing day‐to‐day variability. This makes it hard to determine if the differences in RMR measured using the 2 methods extend beyond RMR variability within each method. Third, despite randomizing the order of testing modes, a greater proportion of the participants were tested using the canopy first. We addressed this issue by comparing the difference in RMR between the two methods across the order categories and found no differences in ∆RMR between participants who were tested first using the canopy or the mask. Fourth, our results show poor agreement while strong parallel test reliability in RMR between the two methods. Unfortunately, we did not include a third method of reference that does not depend on indirect calorimetry, namely direct calorimetry (Kenny et al., 2017), to validate RMR in our sample, nor did we perform an accuracy evaluation of our metabolic system using ethanol burning procedure (Delsoglio et al., 2020). For this reason, we cannot determine with certainty which of the two methods is more accurate.

## 5 CONCLUSION

The preferred method for measuring RMR is indirect calorimetry using a ventilated canopy system. We report here that RMR measured using a facemask showed poor agreement with and significantly underestimated RMR measured sequentially using a ventilated canopy in the same metabolic system, despite a strong parallel test reliability between the two methods.

We conclude that the non‐ventilated silicone facemask cannot produce comparable metabolic data and should not be used for RMR measurement in place of an air‐dilution ventilated canopy.

Future experiments should compare the test–retest reliability between the two methods, assess their accuracy, and validate the two methods against a third reference method for RMR measurement, preferably direct calorimetry. There is also a need to identify the source of measurement error in the mask and come up with strategies to minimize measurement error in this testing method. This will enable us to determine which method is superior and preferable for measuring RMR in humans, but it is beyond the scope of this paper.

## AUTHOR CONTRIBUTIONS

**Yair Pincu:** Conceptualization; data curation; formal analysis; investigation; methodology; project administration; supervision. **F. N. C. Sakshi:** Data curation; investigation. **Grant A. Chesbro:** Formal analysis; methodology. **Akram Falahati:** Formal analysis; investigation. **Rebecca D. Larson:** Methodology; supervision.

## FUNDING INFORMATION

This work was not supported by any research grant.

## CONFLICT OF INTEREST STATEMENT

The authors declare no conflicts of interest.

## Data Availability

The data that support the findings of this study are available from the corresponding author upon reasonable request.
